# Detection of *Mycobacterium* *tuberculosis* DNA in CD34 ^+^ peripheral blood mononuclear cells of Ugandan adults with latent infection: a cross-sectional and nested prospective study

**DOI:** 10.12688/aasopenres.13108.1

**Published:** 2020-07-29

**Authors:** Jonathan Mayito, Irene Andia Biraro, Stephen T. Reece, Adrian R. Martineau, David P. Kateete

**Affiliations:** 1 Department of Immunology and Molecular Biology, School of Biomedical Sciences, Makerere University College of Health Sciences, Kampala, +256, Uganda; 2 Department of Internal Medicine, School of Medicine, Makerere University College of Health Sciences, Kampala, +256, Uganda; 3 Immunology, MRC/UVRI & LSHTM Uganda Research Unit, Entebbe, +256, Uganda; 4 Department of Medicine, University of Cambridge, Cambridge, CB2 0QQ, UK; 5 Institute of Population Health Sciences, Barts and The London School of Medicine and Dentistry, Queen Mary University of London, London, E1 2AD, UK

**Keywords:** Latent tuberculosis infection, blood biomarker, hematopoietic stem cells.

## Abstract

**Background**: Tuberculin skin test and interferon gamma release assay (IGRA) show limitations in diagnosing latent tuberculosis infection (LTBI) and poorly predict progression to active tuberculosis. This study will explore detection of *Mycobacterium tuberculosis* ( *M.tb*) DNA in CD34 ^+^ peripheral blood mononuclear cells (PBMCs) as a biomarker for LTBI and monitoring chemoprophylaxis response.

**Methods: **In a cross-sectional study, 120 household contacts (60 HIV positive and 60 HIV negative) will be recruited. Also, 10 patients with sputum positive pulmonary tuberculosis and 10 visitors from low incidence countries with no history of TB treatment will be recruited as positive and negative controls, respectively. Participants will donate 100 ml (50 ml for TB patients) of blood to isolate PBMCs using density gradient centrifugation. Isolated PBMCs will be separated into CD34 ^+ ^and CD34 ^-^ enriched cellular fractions. DNA from each fraction will be purified, quantified and subjected to droplet digital PCR targeting *IS6110* (a *M.tb* Complex multi-copy gene) and *rpoB*, a single copy gene. Also, 4 ml of blood will be drawn for IGRA. In a nested prospective study, 60 HIV positive participants will be given 300 mg of Isoniazid Preventive Therapy (IPT) daily for six months, after which they will donate a second 100 ml blood sample that will be processed as described above.

Data from the cross-sectional study will be analysed to determine the proportion of individuals in whom *M.tb* DNA is detectable in CD34 ^+^ and CD34 ^-^ fractions and number of *M.tb* genomes present. Data from the prospective study will be analysed to compare the proportion of individuals with detectable *M.tb* DNA in CD34 ^+ ^and CD34 ^-^ fractions, and median *M.tb* genome copy number, post vs pre-IPT.

**Discussion: **This study will determine whether detection of *M.tb* DNA in CD34 ^+^ PBMCs holds promise as a biomarker for LTBI and monitoring chemoprophylaxis response.

## Abbreviations

BCG, Bacillus Calmette-Guerin; CD, Cluster Of Differentiation; CFP-10, Culture Filtrate Protein-Ten; ELISPOT, Enzyme Linked Immunospot; ESAT-6, Early Secretory Antigenic Target-Six; HIV, Human Immunodeficiency Virus; IGRAS, Interferon Gamma Release Assays; IPT, Isoniazid Preventive Therapy; LTBI, Latent Tuberculosis Infection; LT-pHSC, Long Term Pluripotent Hematopoeitic Stem Cells; MACS, Magnetic Activated Cell Sorting; PBMC, Peripheral Blood Mononuclear Cells; PCR, Polymerase Chain Reaction; TST, Tuberculin Skin Test; UNCST, Uganda National Council Of Science And Technology

## Introduction

Tuberculosis (TB) is the leading cause of death from a single infectious agent, having killed 1.5 million HIV negative and 251,000 HIV positive individuals in 2018 ^1^. In 90% of exposed individuals, TB infection becomes contained as latent ^2^, and majority of active disease (47–87%) in low-prevalence settings is due to reactivation of the latent TB infection (LTBI) ^3, 4^. Consequently, an estimated 1.7 billion people in the world have LTBI ^5, 6^, 5% progress to active TB disease within 18 months and 15% progress to active TB disease within their lifetime ^7– 9^. Untreated HIV-positive individuals are 20 times more likely to develop LTBI, and have a 10% risk per year ^9, 10^, and a 30% lifetime risk of progressing to active TB compared to 10% for HIV-negative individuals ^11^.

Due to lack of a gold standard, LTBI diagnosis is based on absence of symptoms or signs of active disease, a normal chest X-ray, and a positive tuberculin skin test (TST) or interferon gamma release assay (IGRA) ^12^. The TST is based on the delayed type hypersensitivity reaction to a purified protein derivative ^13^. An induration of more than 5 mm in low incidence settings and HIV positive individuals, and more than 10 mm in high incidence settings and HIV negative individuals, read after 48 to 72 hours, is considered reactive ^14^. However, an induration of 15 mm or greater is more predictive of progression to active TB within two years ^15^. On the other hand, the TST cannot differentiate between immunological sensitisation to environmental mycobacteria or *M.tb* infection ^16, 17^. The TST also has a low positive predictive value (PPV) of 1.5-2.1% for progression to active TB ^18^. Also, false negative results can be due to T cell anergy, malnutrition, immuno-suppression, incorrect administration of the test or false positive results due to Bacillus Calmette-Guerin (BCG) vaccination ^19, 20^. On the other hand, IGRA measures the quantity of interferon-γ (QuantiFERON-TB Gold In-Tube) or quantity of interferon-γ-producing cells (T-SPOT) after incubation of an individual’s whole blood to *M.tb* synthetic peptides which are absent from most non-tuberculous mycobacterial species. The QuantiFERON-TB Gold In-Tube uses peptides from ESAT-6 (early secretory antigen-6), CFP-10 (culture filtrate protein-10), and TB7.7. The T-SPOT test uses peptides from ESAT-6 and CFP-10 only ^13^. Like TST, IGRAs also have a low PPV (2.7%) for progression to active TB ^21, 22^, and therefore around 100 individuals need to be treated with chemoprophylaxis to prevent one case of TB, a situation not feasible nor cost effective for scaling up LTBI treatment in resource limited settings ^23^.

On the other hand, hematopoietic and mesenchymal stem cells show a tendency to harbour *M.tb* in humans and are an emerging focus of research on the diagnosis of LTBI. Properties of hematopoietic and mesenchymal stem cells that are favourable for intracellular *M.tb* infection include; residence in an immune privileged and hypoxic environment that protects *M.tb* from immune attack and promotes dormancy ^24– 26^, possession of drug efflux pumps that protect *M.tb* from the antibiotic effects, lack of anti-mycobacterial mechanisms in these cells and ability to transition from residency in the bone marrow (BM) into the peripheral circulation to disseminate and potentially interact with developing TB granulomas ^27– 29^. Das *et al.* ^30^ show that BM mesenchymal stem cells (CD271 ^+^/CD45 ^−^ BM MSCs) from healthy volunteers could be infected by *M.tb*. The *M.tb* remained viable and continued to replicate slowly without impeding the development of the CD271 ^+^ BM-MSCs, and infected MSCs were present in a mouse model of dormant TB ^30^. Tornack *et al*. ^31^ showed that long term pluripotent hematopoietic stem cell (LT-pHSCs) harvested from the blood of IGRA positive individuals contained *M.tb* that expressed dormancy-associated genes. In this setting, *M.tb* contained in LT-pHSCs did not readily form colonies on solid mycobacterial growth medium, but were culturable after intratracheal administration to immune-deficient mice where nascent lung granulomas were formed. Furthermore, Reece *et al*. ^32^ showed that murine hematopoietic stem and progenitor cells containing *M.tb* propagated TB infection when transferred to naive mice when both transferred BM cells and the recipient mice were unable to express inducible nitric oxide synthase (NOS2). NOS2 mediates killing of intracellular bacteria via production of nitric oxide but NOS2 is not expressed in resting cells including HSCs ^33^.

The proposed study aims to evaluate detection of *M.tb* DNA in CD34 ^+^ PBMCs as a biomarker for LTBI and for monitoring the response to isoniazid preventive therapy (IPT). Detection of *M.tb* DNA in the CD34 ^+^ PBMCs has potentially better specificity and higher PPV than TST or IGRA, and would therefore allow more targeted chemoprophylaxis in individuals with LTBI. This would improve TB control through targeting of chemoprophylaxis to those who require it.

The study will evaluate presence of *M.tb* DNA in CD34 ^+^ PBMC cell fractions as a biomarker for LTBI as opposed to the TST and IGRA, which are based on detection of *M.tb*-specific immune response. Because presence of *M.tb* DNA in the CD34 ^+^ PBMC cell fraction could represent a microbiological marker of infection, it has potential for better sensitivity and specificity of LTBI diagnosis. Magnetic activated cell sorting (MACS) rather than fluorescent activated cell sorting (FACS) will be used for isolation of cell fractions. Although FACS generally produces purer cellular populations, the MACS procedure utilised here has been optimised and consistently generated a yield of CD34 ^+^ cells that was comparable to that generated by FACS. In addition, MACS is a more cost-effective method of cell isolation and can easily be deployed in resource limited settings.

The specific study aims are as follows:

1. To determine whether *M.tb* DNA is detectable in CD34 ^+^ PBMCs of household contacts of index tuberculosis patients without clinical or radiological features of active disease.
2. To determine whether IPT reduces the proportion of CD34 ^+^ PBMCs with *M.tb* DNA and reduces *M.tb* genome copies in CD34 ^+^ PBMCs of HIV positive household contacts of index tuberculosis patients in whom it was detected at the baseline.
3. To determine the prevalence and determinants of IGRA positivity among household contacts of index TB patients with a negative WHO symptom screen.

The hypotheses underpinning the study objectives are grounded in relational pathways of selective risk factors that interact with the main exposures and outcomes as depicted in the conceptual framework ( *Extended data:* Appendix I ^34^). In this framework, the host risk factors (distal factors) increase the risk for TB infection. The risk factors for CD34 ^+^ PBMCs infection by *M.tb* (proximal factors) are grounded in the presence of a quiescent BM environment for *M.tb* persistence. The infected CD34 ^+^ PBMCs can pass the BM barrier into peripheral blood circulation and have the potential to propagate TB.

## Methods

### Study design

****Study one:**** a comparative cross-sectional study carried out among HIV positive versus HIV negative household contacts (HHCs) of index TB cases, to compare the proportions of participants that have detectable *M.tb* DNA in CD34 ^+^ PBMCs.

****Study two:**** a prospective study carried out among HIV positive HHCs during 6 months of IPT to determine whether IPT reduces the prevalence of detectable *M.tb* DNA in CD34 ^+^ PBMCs and the number of *M.tb* genome copies.

****Study three:**** a cross-sectional study carried out to determine the prevalence and determinants of IGRA positivity among HHCs of index TB patients.

The Arkansas colorimetric test for measuring adherence to IPT will not be used. Instead, participants will be reminded to take their medication via a monthly telephone call. Adherence will be assessed through pill count at each study visit.

### Study sites

Participants will be recruited from the Mulago National Referral Hospital, Infectious Diseases Institute (IDI; Kampala, Uganda) and Kisenyi Health Centre IV TB clinics (Kisenyi Health Centre IV). The PBMC isolation and subsequent CD34 ^+^ and CD34 ^-^ cell fraction isolation will be carried out at the IDI Translational Research Diagnostic Laboratory, while DNA extraction from CD34 ^+^ and CD34 ^-^ PBMCs fractions will be carried out at Queen Mary University of London (QMUL) Biological and Chemical Sciences Laboratory (London, UK). DNA samples will be subjected to ddPCR at Hutchison/MRC Research Centre Laboratory of Molecular Biology (LMB; Cambridge, UK), University of Cambridge (UC) or the Local Government Chemist Laboratory (LGC; Teddington, UK).

### Study eligibility criteria

The study population will be HIV-positive and HIV-negative HHCs of sputum positive (smear or GeneXpert) pulmonary TB (PTB) patients. They will be selected according to the criteria below.

***Inclusion criteria***

1. Household contact of an index TB patient, who has been living with the patient while the patient was symptomatic before initiating treatment;
2. Has no clinical (negative TB symptom screen) or radiological features (normal chest X-ray (CXR)) of active TB;
3. Aged 18 years and above;
4. Able to give written informed consent;
5. Contactable by telephone and able to attend a follow-up visit.

***Exclusion criteria***

1. Anyone who ever received IPT or standard anti-mycobacterial treatment;
2. Any woman who is pregnant or suspects that they are pregnant and cannot therefore take a CXR for safety of the baby;
3. Anyone with a medical condition which in the opinion of the investigator may interfere with the participant’s ability to participate in the study.

***Inclusion criteria for controls***

*Positive controls*: smear or GeneXpert positive PTB patients who have not yet initiated anti-tuberculous treatment.

*Negative controls*: visitors from a TB low burden country without any history of contact with a pulmonary TB patient or history of being treated for TB.

### Study one

***Screening procedures***

The HHCs will be briefed and invited to consent for screening, as described below:

1. *World Health Organization (WHO) four symptom screening:* cough of any duration, weight loss, fever and night sweat. Those that have a negative screening result will proceed into the study. Those with a positive screening result will undergo further TB investigation.
2. *Physical examination*: weight, height, blood pressure, peripheral lymphadenopathy, palpable abdominal masses, joint swelling and vertebral column gibbus. Anyone with the above and other features of extra-pulmonary TB will undergo further TB assessment, while those without will proceed into the study.
3. *CXR:* those with a normal CXR will proceed in the study while those with an abnormal CXR will undergo further TB screening.
4. *HIV-1 and HIV-2 screening test using the national testing algorithm*: both HIV-positive and HIV-negative individuals will proceed in the study, and those positive linked to HIV care.

***Enrolment procedures***

All eligible HIV-positive and HIV-negative individuals will be enrolled into the study following the procedures below:

1. Assessment of social demographics, bacillary load of the index case’s sputum, relationship, level of intimacy, and duration of contact with index patient, smoking history and presence of a BCG scar.
2. Donation of 100 ml of whole blood for isolation of PBMCs, isolation of CD34 ^+^ and CD34 ^-^ PBMC fractions, purification of DNA from PBMC fractions and subsequent detection of the *M.tb* DNA, and 4 ml blood for IGRA.
  100 ml of blood is required because CD34 ^+^PBMCs are present in peripheral blood in a small proportion (0.01%). 100 ml of blood yields 1-5 x 10 ^5^ CD34 ^+^ cells, of which approximately 10 ^3^ are CD34 ^+^ PBMCs. The CD34 ^+^ PBMCs may harbour 1-100 *M.tb* genomes. Therefore a 100 ml blood draw gives a reasonable chance of obtaining the required yield of LT-HSCs within the CD34 ^+^ fraction *.*

All HIV-negative participants will exit the study at this point while the HIV-positive participants will be consented for study two.

3. Ten healthy visitors from low TB incidence countries with no history of contact with TB patients or treatment for TB (negative controls), and 10 index PTB smear/GeneXpert positive participants who have not yet initiated TB treatment (positive controls) will be recruited and requested to donate 100 ml and 50 ml of blood, respectively, for the procedures described above. These controls will support validation of the study results, i.e. the detection or failure to detect *M.tb* DNA in CD34 ^+^ PBMCs.

### Study two

***Enrolment procedures.*** The HIV-positive participants who took part in study one will be consented for study two. All consenting participants will undergo adherence counselling before being initiated on 300 mg of isoniazid and 25 mg of pyridoxine daily for 6 months. They will be given three monthly refills but will be telephoned every month to remind them to take the medication. Adherence will be assessed by pill count at each study visit. At the end of 6 months, they will be requested to donate another 100 ml of blood for PBMC isolation, CD34 ^+^ and CD34 ^-^ PBMC isolation, and extraction and quantification of DNA. They will subsequently exit the study.

### Study three

***Screening procedures.*** HHCs of index TB patients will be identified and consented to participate in study three (including those who took part in study one). They will be screened using WHO TB symptom screening; those who are negative will proceed into the study while those who are positive will undergo further TB screening.

***Enrolment procedures***

1. *Medical history and physical examination:* weight, height, and peripheral lymphadenopathy, palpable abdominal masses, joint swelling and vertebral column gibbus.
2. *HIV-1 and HIV-2 screening using the national testing algorithm:* both HIV-infected and HIV-uninfected will participate in the study and will donate 4 ml of blood for IGRA and then exit the study.

### Study outcomes

***Study one:*** The outcome will be “detection of *M.tb* DNA in CD34 ^+^ PBMCs” as a biomarker for LTBI, and the secondary outcome will be “number of *M.tb* genome copies” among HHCs of index TB patients.

***Study two*:** The outcome will be the “reduction in prevalence of *M.tb* DNA and number of *M.tb* genome copies in CD34 ^+^ PBMCs” following 6 months of IPT among HIV positive HHCs.

***Study three:*** The outcome will be “IGRA positivity rate” and “factors associated with IGRA positivity” among HHCs of index TB patients.

Timelines for the study are shown in Table 1. A study flow chart can be found in *Extended data:* Appendix II ^34^.

**Table 1.** Timelines.

Event	Baseline	Month 3	Month 6
***Study one***			
Consent	X		
Screening: *Symptom & CXR*	X		
Enrolment:	X		
History: *Cough, fever, weight loss & night* *sweats;* and physical exam: *General,* *respiratory, abdominal, spine & joint*	X		
IGRA test – *4ml*	X		
CD34 ^+^ PBMC *M.tb* DNA test – *100ml*	X		
Study exit if HIV negative	X		
***Study two***			
Enrolment of HIV positive	X		
History: *Cough, fever, weight loss & night* *sweats;* and physical exam: *General,* *respiratory, abdominal, spine & joint*	X		X
IGRA test – *4ml*	X		
CD34 ^+^ PBMC *M.tb* test – *100ml*	X		X
Drug pick up: * Isoniazid 300 mg & Pyridoxine 25 mg*	X	X	
Adherence assessment: Pill count		X	X
Study exit			X
***Study three***			
Consent	X		
Screening: *Symptom of HHCs*	X		
Enrolment of symptom negative HHCs	X		
History: *Cough, fever, weight loss & night* *sweats;* and physical exam: *General,* *respiratory, abdominal, spine & joint*	X		
IGRA test – *4ml*	X		
Study exit	X		

### Sample size

***Study one:*** the main comparisons of the study outcome will be between HIV-positive versus HIV-negative, and IGRA positive versus IGRA negative participants. The sample size formula for comparing two-proportions was used ^35^:

$$\text{n}=({\text{Z}}_{\alpha /2}+{\text{Z}}_{\text{β}})2*(\text{p}1(1-\text{p}1)+\text{p}2(1-\text{p}2))/\;(\text{p}1-\text{p}2)2$$

P1 and P2 are the proportions among HIV-positive and HIV-negative respectively, expected to have detectable *M.tb* DNA in CD34 ^+^ PBMC cell fractions. We estimate that about 30% of the HIV-positive and 10% of HIV-negative participants are likely to develop active TB in their life time and thus may be the proportions harbouring *M.tb* ^11^. We therefore assume that P1=30% and P2=10%.

Zα _/2_ is the critical value of the normal distribution at α/2 (for a confidence level of 95%, α is 0.05 and the critical value is 1.96), and Z _β_ is the critical value of the normal distribution at β (for a power of 80%, β is 0.2 and the critical value is 0.84).

Based on the above formula, the sample size required at 5% level of significance and 80% power is 59 for each group and 118 in total.

The sample size of 59 participants per group has been inflated by 1 participant to allow for an adequate sample size for the follow up study. Therefore, the sample size will be 60 HIV-positive and 60 HIV-negative, and overall 120 participants. In addition, 10 participants with smear/ GeneXpert positive PTB and, 10 participants from low TB burden countries with no TB symptoms or prior TB treatment will be purposively recruited to function as positive and negative controls, respectively.

****Objective two:**** sample size calculation was based on the before-after (Paired T-test) formula below ^36^:

N=A∗(Zα+Zβ)^2(E/S(Δ))^2

Where;

α (two-tailed) = 0.05 - *Threshold probability for rejecting the null hypothesis. Type I error rate.* β = 0.2 - *Probability of failing to reject the null hypothesis under the alternative hypothesis. Type II error rate.* E = *Effect size,* and S (Δ) = *Standard Deviation of the CHANGE in the outcome.*

Based on the findings of Tornack *et al.,* we assume that the mean number of copies of the *M.tb* genome at baseline is 4.3 in PCR-positive participants, with a standard deviation of ±2.5 copies ^31^. Substituting in the above formula, to detect a 25% reduction (E) in mean copy number (from 4.3 to 3.2) with 80% power at the 5% significance level, we would need 41 participants.

Allowing for *M.tb* DNA being undetectable in CD34+ PBMCs in 25% (11) of participants, and taking into consideration a 15% loss to follow-up over 6 months, which is 8, the sample size of 60 participants will be sufficient to answer this question.

***Objective three:*** Body Mass Index (BMI) is strongly associated with TB infection. A high BMI is a predictor of LTBI ^37^ while a low BMI is a predictor for progression to active TB ^38^. From a study in an African setting, 57.5% of individuals with a BMI of less than 18.5 had LTBI compared to 41.2% with a BMI higher than 18.5 ^39^. 

Therefore, the main predictor will be BMI, i.e. the effect of a BMI of greater or less than 18.5 on IGRA positivity. A sample size formula for comparing two proportions was used:

$$\text{n}=({\text{Z}}_{\text{α}/2}+{\text{Z}}_{\beta })2*(\text{p}1(1-\text{p}1)+\text{p}2(1-\text{p}2))/\;(\text{p}1-\text{p}2)2$$

P1 and P2 are the proportions among those with low and normal/high BMI respectively expected to have LTBI. Therefore, we have assumed that P1=57.5%% and P2=41.2%.

Z _α/2_ is the critical value of the normal distribution at α/2 (for a confidence level of 95%, α is 0.05 and the critical value is 1.96), and Z _β_ is the critical value of the normal distribution at β (for a power of 80%, β is 0.2 and the critical value is 0.84). Using the formula above, the sample size required at 5% level of significance and 80% power is 144 per group and a total of 288 participants.

### Recruitment plan

For studies one and two, participants will be identified and recruited from the IDI, Mulago Hospital and Kisenyi Health Centre IV TB clinics. For study three, participants will be recruited from the same clinics with a home visit to the index case carried out if necessary. These strategies will enable the study to recruit the required sample size.

### Data collection methods

Data will be collected using pre-tested case report forms (CRF). The CRF will be piloted on 10% of the sample size in non-potential participants. To ensure data integrity, research assistants will be trained on using the protocol and study instruments. Data check files will be developed for the data entry platform, and data entry will be performed in duplicate. 

### Measurements of study outcomes

***IGRA (QuantiFERON-TB ^®^ Gold-plus)***: The QuantiFeron assay (Cellestis) is a commercial qualitative ELISA assay. 1 ml of blood will be collected in each of the four tubes containing; ESAT-6 (green) and CFP-10 (yellow top), phytohaemagglutinin (purple top) as the positive control, and no antigen in the fourth tube (grey top) as the negative control. The blood will be mixed thoroughly by inverting the tube top-bottom 10 times and tubes will be transported to the laboratory at room temperature within 16 hours from the time of collection. The tubes will then be cultured for 16–24 hours at 37 ^o ^C and then centrifuged at 3000 g. The supernatant will be harvested, stored at -80 ^o^C and tested in batches using the standard QuantiFeron ELISA protocol to determine the amount of interferon-γ using the QIAGEN standard operating procedures. The standards of the ELISA will be run in duplicate while the samples will be run in singlets, a total of 28 tests per ELISA plate.

***Isolation of PBMCs (Extended data: Appendix III ^34^):*** The PBMCs will be isolated from peripheral blood using density gradient centrifugation method based on the principle of differential migration of blood cells through the histopaque solution. Lysis of red blood cells will not be carried out as it could affect the yield of CD34 ^+^ PBMCs. Anti-coagulated blood will be carefully layered onto the histopaque and briefly centrifuged to form cellular layers. The layer containing mononuclear cells together with platelets found at the interface between plasma and histopaque solution will be harvested and washed with a salt solution to remove platelets, histopaque and plasma, and then centrifuged again. The mononuclear layer will then be used for isolation of the CD34 ^+^ PBMCs. 

***Isolation of the CD34 ^+^PBMCs (Extended data: Appendix III ^34^):*** CD34 ^+^ PBMCs will be isolated from the CD34 ^-^ PBMCs using Miltenyi Biotec MACS MicroBeads. The MACS MicroBeads are 50-nm super-paramagnetic particles conjugated to highly specific antibodies against the CD34 ^+^ cell surface marker. PBMCs will be magnetically labelled directly with MACS MicroBeads and the sample applied to a MACS Column placed in a MACS Separator. The unlabelled cells pass through while the magnetically labelled cells (the CD34 ^+^ PBMCs) are retained within the column. After a short washing step, the column will be removed from the separator, and the magnetically labelled cells will be eluted from the column **.**

***Culture of M.tb from the CD34 ^+^ and CD34 ^-^ PBMC cell fractions:*** Aliquots of CD34 ^+^ and CD34 ^-^ PBMC fractions from 10 HIV positive and 10 HIV negative participants will be cultured for *M.tb* in 7H9 / BACTEC MGIT (Mycobacteruim Growth Indicator Tube) before boiling, for 4 to 6 weeks. This will function as a control to validate whether the detected *M.tb* is viable.

***Extraction of DNA from CD34 ^+^ and CD34 ^- ^PBMC cell fractions:*** The extraction of DNA from CD34 ^+^ and CD34 ^-^ PBMC cell fractions will be carried out using the Hexadecyltrimethylammonium bromide (CTAB) protocol ^40^. The protocol involves the use of lysozyme, CTAB/NaCl buffer, protein kinase K, 10% SDS in distilled water, Chloroform/Isoamyl alcohol, isopropanol, 70% ethanol and Tris EDTA. 

***Quantification of M.tb DNA recovered from CD34 ^+^ and CD34 ^-^ PBMC cell fractions:*** *M.tb*-specific DNA sequences will be amplified, and number of genomes quantified by ddPCR using gene-specific primers targeting the *IS6110* (multi-copy) and *rpoB* (single copy) genes. The assays used are sensitive enough to detect one *M.tb* genome.

### Retention and follow-up

Participants for study two will have follow-up. Contact information including telephone number for the participant and next of kin will be collected to enable follow-up. Participants will be called by telephone every month to remind them about taking medication and as a way of maintaining contact with them. They will come to the clinic at three months to collect medication as well as at six months for final procedures and exit from the study. At all study visits, adherence to the study procedures will be emphasized and participants will receive reimbursement for travel. For participants who do not complete follow-up, only their baseline data will be used for analysis of study one.

### Data management

All CRFs will be stored in a lockable cabinet placed in a lockable room. Only authorised study staff will have access to the study data. The data from the CRFs will be entered in duplicate by two different individuals. The second entry will validate the first and any discrepancies will be resolved before the data is uploaded into the database. Data will be identified by subject identification number which will be used in analysis, dissemination and publication. Data will be entered using EpiData 3.1 and exported to STATA statistical software package version 13.0 (StataIC Corporation, College Station, Texas USA) for analysis.

### Predictor variables

The main predictor variable will be HIV status, while the secondary variables will include: IGRA status, BMI, demographic characteristics, relationship with index patient, bacillary load of index patient, level of intimacy with index patient, intensity of contact with the index patient, duration the index patient coughed, smoking history and presence of BCG scar.

### Data analysis

***Descriptive analysis.*** The continuous variables will be reported as means with their standard deviations for normally distributed variables, while the median will be reported for skewed distribution since it is more stable to outliers. The categorical variables will be reported as proportions in terms of frequencies and percentages.

For study one, the diagnostic performance of detection of *M.tb* DNA in CD34 ^+^ PBMCs ** as a biomarker for LTBI will be determined by cross tabulation of the outcome “detection or no detection of *M.tb* DNA in CD34 ^+^ PBMCs” with IGRA status, as indicated in Table 2.

**Table 2.** Diagnostic performance of “Detection of M.tb DNA” against IGRA.

	IGRA positive	IGRA negative	
*M.tb* DNA detected	a	b	a+b
*No detection of M.tb* DNA	c	d	c+d
	a+c	b+c	

***Bivariate analysis.*** The main outcomes of interest for studies one, two and three, will be “detection of *M.tb DNA*”, “reduction in prevalence and *M.tb* genome copies”, and “IGRA positivity”, respectively. The predictor variables will be cross tabulated with the outcome variables and compared using a chi square test to determine if they are statistically different. For study two, where the outcome “reduction in *M.tb* genome copies” is continuous, a paired T test if distribution of number of *M.tb* genome copies is Gaussian, or a Wilcoxon signed rank sum test if the distribution is non-Gaussian, will be done to compare the magnitude of reduction in the *M.tb* genome copies pre versus post IPT. The results will be presented in bivariate tables and will include; the chi square statistic and the P-values. At all comparison, the level of significance to test whether to reject or fail to reject the null hypothesis will be *P*-value of ≤ 0.2.

***Multivariable analysis.*** The main predictor variables for studies one, two and three include HIV status, IGRA status and BMI, respectively. A logistic regression model will be built using main predictor variables and the other predictor variables added to the model based on the level of significance of their odds ratios at bivariate analysis. The predictor variables that are not significant at bivariate, but have biological plausibility to the outcome variables, will also be included in the model. The logical stepwise model building method will be used. At multivariable analysis, the level of significance to test whether to reject or fail to reject the null hypothesis will be a *P*-value of ≤ 0.05.

### Adverse event reporting

At the follow-up visits, the study investigator will assess for adverse events (AEs) that may have occurred since the previous visit. The investigators will generate and submit annual reports summarizing these AEs to the institutional review board and the Uganda National Council of Science and Technology (UNCST). All AEs will be managed according to the standard clinical protocol of IDI and Mulago National Referral Hospital.

### Ethics and consent

***Ethical approval and consent to participate.*** The study protocol, informed consent forms ( *Extended data:* Appendix IV ^34^), CRFs, and recruitment materials have been reviewed and approved by the Makerere University College of Health Sciences’ School of Biomedical Sciences Research and Ethics Committee (SBS-REC; Reference no. SBS 595) and UNCST (Reference no. 2522), prior to implementation of any study activities. Administrative clearance to conduct the study was also provided by Mulago National Referral Hospital, IDI and Kisenyi Health Centre IV. All violations, deviations and amendments will be submitted to the SBS-REC for review and approval respectively.

All participants will give written informed consent before any study related procedure. The study will be explained to each index case and their contacts in a language they can understand, and written consent obtained individually for every participant. Consent will be sought for storage of PBMCs for future TB related research ( *Extended data*: Appendix V ^34^). A copy of the consent forms will be given to the participant and another kept for study records 

***Consent to publish.*** Informed consent is being sought from all participants before any study related procedures are performed. Data from the study will be published in a peer reviewed journal without any personal identification data of the participants.

***Confidentiality assurances.*** The clinical data will be entered into a study specific data base by a designated staff on a regular basis from completed CRFs. All the CRFs will be reviewed daily by the investigator to resolve any missing data or anything else that might warrantee attention. The data base will be updated regularly on a secure computer, as will the data that are received from the laboratory. Access to the database will be given to authorized persons only (members of the immediate study team) and a log of authorized personnel will be stored in the study master file. CRF and study documents will be locked in lockable cabins. No participant identifying information will be disclosed in any publication or at any conference activities arising from this study.

## Dissemination of study outcomes

All study related documents will be kept for at least 5 years and the data from this work will be deposited at a selected open access data repository. The results will be published in high-impact peer-reviewed journal. All the data used in the publication will be anonymized. The results will also be presented at an international conference, to officials of the National TB and Leprosy Program, Ministry of Health, study participants and other policy stakeholders. The sponsors will also receive periodic reports.

## Study status

Recruitment for study one and study three is still ongoing but nearing completion, while recruitment and follow-up from study two has been completed.

## Discussion

This study seeks to address critical gaps in the diagnosis, and monitoring treatment of LTBI. The gaps that will be addressed include: a) inability of TST and IGRA to differentiate between cleared and ongoing LTBI because being based on detection of TB specific immune responses, they may remain positive even when infection has been cleared; and b) lack of a test to monitor response to LTBI treatment because the TST and IGRA have low reversion rates.

The study is in line with the END TB Strategy’s goal of finding innovative ways of screening contacts and high-risk groups so that they are targeted for treatment, to aid the elimination TB as a public health problem by 2035. It will explore the presence of *M.tb* DNA in CD34 ^+^ PBMCs as a microbiological biomarker for LTBI with potential for having better specificity than TST or IGRA. A biomarker with better specificity will ensure that only individuals at increased risk of developing active TB are targeted for preventive therapy. Targeting preventive therapy to only those with increased risk of active TB would make the scale up of preventive therapy more cost effective and attractive for resource limited settings where uptake has been slow among groups in whom it has been recommended.

Hematopoietic stem cells present within CD34 ^+^ PBMC cell fractions represent a potential niche for *M.tb* during latency due to these cells lacking anti-mycobacterial killing mechanisms, their habitation in a quiescent and immune privileged environment in the BM, and their possession of active drug efflux pumps. Because of these properties, the *M.tb* present in CD34 ^+^ PBMCs avoids adverse conditions including hypoxia, immune attack and drug therapy, to allow persistence for long periods in the host. For these reasons, interrogating these cells for the presence of *M.tb* for the diagnosis of latent TB and monitoring success of preventive treatment could provide valuable insights.

Our study has some limitations. Use of MACS means the cell fractions we analyse will contain a small proportion of contaminating cell types. To mitigate against this, our MACS method has been optimised and consistently generated a yield of CD34 ^+^PBMCs that was comparable to that generated by FACS, a more accepted method to produce pure cellular populations. MACS was selected for this study as a more cost-effective method than FACS and is more easily deployed in resource limited settings. Our study will also not involve following up participants found to have detectable *M.tb* DNA in their CD34+PBMCs and therefore will not determine the predictive potential of the biomarker for progression to active TB.

In summary, this study will determine whether *M.tb* DNA can be detected in CD34 ^+^ PBMCs of asymptomatic adults living in Uganda, and if so, whether IPT can ablate or attenuate this signal. It is hoped that the results, be they positive or negative, will provide insights with potential to inform development of microbiological biomarkers of LTBI.

## Data availability

### Underlying data

No data is associated with this article.

### Extended data

Open Science Framework: Detection of Mycobacterium tuberculosis DNA in CD34 ^+^ peripheral blood mononuclear cells of Ugandan adults with latent infection: A cross-sectional & nested prospective study, https://doi.org/10.17605/OSF.IO/6W5DU ^34^. This project was registered on 15 ^th^ July 2020 ( https://osf.io/vufqa).

This project contains the following extended data:

- Appendix I: Conceptual framework
- Appendix II: Study flow chart
- Appendix III: PBMC isolation and CD34+ PBMC sorting SOP
- Appendix IV: Informed consent form
- Appendix V: Blood storage consent form

Data are available under the terms of the Creative Commons Zero "No rights reserved" data waiver (CC0 1.0 Public domain dedication).
