# Supplementary material for: Detection of Mycobacterium tuberculosis DNA in CD34 + peripheral blood mononuclear cells of Ugandan adults with latent infection: a cross-sectional and nested prospective study
Source: AAS Open Res. 2020 Jul 29;3:34. [Version 1] doi: 10.12688/aasopenres.13108.1 (PMC7422845; doi:10.12688/aasopenres.13108.1)
Supplement: Peer review history

# Peer review: Reviewer response for version 1

**DOI:** 10.21956/aasopenres.14208.r27717

**Published:** 2020-08-11

**Authors:** Masaki Yamamoto

**Affiliations:** 1 Respiratory Disease Center, Yokohama City University Medical Center, Yokohama, Japan

The manuscript "Detection of Mycobacterium tuberculosis DNA in CD34+ peripheral blood mononuclear cells of Ugandan adults with latent infection: a cross-sectional and nested prospective study" reports a study protocol exploring detection of Mycobacterium tuberculosis (M.tb) DNA in CD34+ peripheral blood mononuclear cells (PBMCs) as a biomarker for LTBI and monitoring response to isoniazid preventive therapy (IPT), because several lines of evidence from human and animal model studies showed that hematopoietic stem cells contained M.tb. The aims of this study are follows.

1. To determine whether M.tb DNA is detectable in CD34+ PBMCs of household contacts of index tuberculosis patients without clinical or radiological features of active disease.
2. To determine whether IPT reduces the proportion of CD34+ PBMCs with M.tb DNA and reduces M.tb genome copies in CD34+ PBMCs of HIV positive household contacts of index tuberculosis patients in whom it was detected at the baseline.
3. To determine the prevalence and determinants of IGRA positivity among household contacts of index TB patients with a negative WHO symptom screen.

The study design for the first aim, a cross-sectional study among HIV positive versus negative household contacts (HHCs) of index TB cases, is planned to compare the proportions of participants that have detectable M.tb DNA in CD34+ PBMCs. For the second aim, a prospective study among HIV positive HHCs after 6 months of IPT is designed to determine whether IPT reduces the prevalence of detectable M.tb DNA in CD34+ PBMCs and the number of M.tb genome copies. For the third aim, a cross-sectional study is designed to determine the prevalence and determinants of IGRA positivity among HHCs of index TB patients.

 As a better biomarker for LTBI and for monitoring the response to isoniazid preventive therapy (IPT) is demanded in all over the world, the aims of this study are interesting for researchers in this field.

 The proposed protocols, data managements, ethical considerations are almost agreeable. It would be ethically unacceptable, but a prospective study among HIV positive HHCs without IPT would be appropriate to evaluate the efficacy of IPT. Participants with poor adherence could be substitutes for this intent.

Is the study design appropriate for the research question?

Partly

Is the rationale for, and objectives of, the study clearly described?

Yes

Are sufficient details of the methods provided to allow replication by others?

Yes

Are the datasets clearly presented in a useable and accessible format?

Yes

Reviewer Expertise:

respiratory diseases, especially infectious diseases.

I confirm that I have read this submission and believe that I have an appropriate level of expertise to confirm that it is of an acceptable scientific standard.

# Peer review: Reviewer response for version 1

**DOI:** 10.21956/aasopenres.14208.r27716

**Published:** 2020-08-10

**Authors:** Emmanuel O. Babafemi

**Affiliations:** 1 School of Pharmacy and Biomolecular Sciences, Liverpool John Moores University, Liverpool, UK

The rationale for and objectives of the study are clearly stated and aligned with the strategy to end the global TB epidemic by 2035.

 The study design of the cross-sectional and nested prospective study with relevant compartmentalising different stages into study one - a comparative cross-sectional among HIV+ & HIV- HHCs, study two-prospective studies for carrying out whether IPT reduces the prevalence of detectable *M.tb* DNA in CD34+ PBMCs and the number of *M.tb* genome copies among HIV+ HHCs and study three - a cross-sectional study to determine the prevalence and determinants of IGRA positivity among the HHCs of index TB patient is appropriate for the research question.

 There are sufficient and transparent details of the research methods that allow for replication by other researchers in the field.

 The proposed data management plan and standard software packages are robust for a research study of this magnitude.

Is the study design appropriate for the research question?

Yes

Is the rationale for, and objectives of, the study clearly described?

Yes

Are sufficient details of the methods provided to allow replication by others?

Yes

Are the datasets clearly presented in a useable and accessible format?

Yes

Reviewer Expertise:

Infectious disease with a special interest in Mycobacterium tuberculosis diagnosis using molecular detection

I confirm that I have read this submission and believe that I have an appropriate level of expertise to confirm that it is of an acceptable scientific standard.

# Response

**Published:** 2020-08-10

**Authors:** Jonathan Mayito

**Affiliations:** Makerere University, Uganda

Thank you Emmanuel, for your time to review this article and the positive comments. Thank you too for having the same enthusiasm about this work that seeks to bridge the gap in the diagnosis of latent tuberculosis through evaluating CD34+PBMCs as target cells for detecting M.tb during latency.
